# Computing threshold antibody levels of protection in vaccine clinical trials: An assessment of methodological bias

**DOI:** 10.1371/journal.pone.0202517

**Published:** 2018-09-07

**Authors:** Merryn Voysey, Manish Sadarangani, Andrew J. Pollard, Thomas R. Fanshawe

**Affiliations:** 1 Nuffield Department of Primary Care Health Sciences, University of Oxford, Oxford, United Kingdom; 2 Oxford Vaccine Group, Department of Paediatrics, University of Oxford, Oxford, United Kingdom; 3 NIHR Oxford Biomedical Research Centre, Oxford, United Kingdom; 4 Vaccine Evaluation Center, BC Children’s Hospital Research Institute, University of British Columbia, Vancouver, Canada; Public Health England, UNITED KINGDOM

## Abstract

In the development of new vaccines, understanding the level of vaccine-induced antibody that is sufficient to protect against disease can simplify and expedite the development and licensing process. If there is an accepted threshold antibody level that is indicative of protection, then smaller trials measuring antibody concentration alone can be conducted to test new vaccines, instead of large efficacy studies powered on clinical outcomes. Commonly, threshold levels of protective antibody are determined from clinical efficacy trials in which clinical endpoints are measured on everyone and a small subset of participants have antibody concentrations measured. The proportion of participants with antibody below a threshold in each group in the immunogenicity subset can be compared to the proportions with disease in each group in the larger trial to find an appropriate threshold. Mathematically, this method seeks to compute an absolute threshold whereby antibody above the threshold provides complete, sterilizing immunity. However, in practice it is often understood that such thresholds may only be indicative of a relative degree of protection rather than an absolute one. Although this approach is common, the accuracy of such methods when the underlying mathematical assumptions do not hold true, has never been tested. We simulated data from clinical trial scenarios under varying assumptions of vaccine efficacy and calculated antibody thresholds of protection. We estimated the bias in the calculated thresholds derived from each scenario and showed that in many situations this method produces inflated estimates of thresholds, particularly if a vaccine induces high levels of antibody or when the underlying assumption of sterilizing immunity is violated.

## Introduction

The process of developing, testing, and licensing a new vaccine necessitates an assessment of the vaccine’s ability to prevent disease in the population of interest. This can be done by conducting clinical trials in which participants are randomised to receive the new vaccine or a placebo/control vaccine, and are subsequently followed up for a period of time to determine disease outcomes. Randomised placebo-controlled clinical efficacy trials have been used to assess many vaccines including the 7-valent pneumococcal vaccine, [1–3] rotavirus vaccine, [4, 5] typhoid, [6, 7] cholera, [8] and many others. If clinical outcomes in such trials are hard to measure due to tests that lack sensitivity or other difficulties in diagnosis (e.g. blood culture positive typhoid fever, invasive pneumococcal disease), then outcomes may occur in a relatively small proportion of participants. Such trials can require tens of thousands of participants, and involve high costs, and a substantial number of years investment. If an immunological parameter (a ‘correlate of protection’) can be identified that is associated with protection against disease, such as circulating antibody, neutralising antibody, or a measure of cellular immunity, then the process of testing a new vaccine can be accelerated. Smaller trials of hundreds rather than thousands of participants can then be conducted, in which immunological measures are assessed rather than clinical disease endpoints.

The 10-valent and 13-valent pneumococcal conjugate vaccines were licensed using immunogenicity trials alone. No clinical efficacy data were available for the vaccines however clinical efficacy trials had been conducted for the 7-valent vaccine and these informed the derivation of an overall threshold (across all vaccine serotypes) that was used in licensing the newer vaccines.[9] There were three pneumococcal conjugate vaccine efficacy trials used to assess the antibody correlate of protection, which when combined included 65 834 children. There were, however, only 61 cases of vaccine-type invasive pneumococcal disease (0.09%), and 1863 (2.8%) children had blood samples taken for antibody measures.[10] Such low proportions mean that the children sampled for immunogenicity and the children with disease were essentially different groups of children and no statistical models can be fit to these data to link the antibody responses to disease outcomes on an individual participant level. A post-licensure study was used to refine the previous overall estimate of the threshold correlate of protection for the 13-valent vaccine. Thus was done in the UK based on a large dataset of 716 serotyped cases on invasive disease. The larger sample size allowed for estimates to be made on a serotype-specific level.[11]

Group C meningococcal conjugate vaccines were also licensed in the UK based on serological assessment of efficacy without clinical efficacy studies of the conjugate vaccine.[12] The correlate of protection used for licensing had been previously established based on studies of group C polysaccharide vaccine [13] and subsequently validated for the conjugate vaccine using postlicensure surveillance data.[14] After introduction of these vaccines into the routine infant immunisation programme there were substantial decreases in disease incidence, confirming the clinical efficacy of these vaccines.

For other vaccines such as those for typhoid, rotavirus, and ebolavirus, there is no established correlate of protection and no thresholds that can be used to predict efficacy of a new vaccine. For these vaccines it is therefore still necessary to conduct large field efficacy studies to establish efficacy.

To determine the level of antibody that is sufficient to protect against disease, one needs data on disease events as well as antibody measures in those vaccinated and a way of correlating these two entities. When regression models cannot be applied, an alternative method is used in which the proportion of participants with antibody below a threshold in each randomised group in the immunogenicity subset is compared to the proportions with disease in each group in the larger trial.

In this paper we examine this alternative model commonly used to define a threshold cutpoint of the correlate of protection. This method was successfully used to license pneumococcal and meningococcal vaccines in recent decades without large clinical efficacy studies of the new vaccines. We simulate scenarios based on both high and low efficacy vaccines, with varying relationships between the immunological measure and the likelihood of disease, and explore the performance of the model in each setting.

Our purpose was to assess the settings in which the method is fit for purpose and to demonstrate situations where the method should be avoided.

## Methods

### Definitions

We use the term ‘correlate of protection’ in this article to describe a measureable immunological parameter induced by vaccination that is related to protection against a specific clinical endpoint. The term ‘threshold of protection’ is used to describe a cutpoint on the correlate of protection that is used to test and compare vaccines, and above which a certain degree of protection is thought to exist. This can be absolute protection (100%) or relative (< 100%) and is affected by the rate at which the level of the immunological parameter wanes post-vaccination. We consider thresholds for correlates of protection measured at 4–6 weeks after vaccination only, as this is the most common and relevant time point for vaccine clinical trials, but recognise that thresholds of protection at other time points, such as the time at which exposure occurs, are also of interest to researchers, and are far more difficult to define and measure.

### Calculating a threshold of protection

The method for calculating an immunological threshold for protection in this simulation study is that which was used to establish thresholds for pneumococcal and group C meningococcal vaccines.[9–11, 14]

Firstly, the observed vaccine efficacy (*VE*.*obs*) is calculated from an efficacy trial or post-licensure study as,

$$VE.obs=1-\frac{\text{Attack}\;\text{rate}\;\text{in}\;\text{vaccine}\;\text{group}}{\text{Attack}\;\text{rate}\;\text{in}\;\text{control}\;\text{group}}$$

where the attack rate is the proportion of participants with clinical disease.

The threshold (*t*) is the calculated from a smaller immunogenicity substudy within the main trial, assuming:

$$VE.ims=1-\frac{\text{Proportion}\;\text{with}\;Ab<t\;\text{in}\;\text{vaccine}\;\text{group}}{\text{Proportion}\;\text{with}\;Ab<t\;\text{in}\;\text{control}\;\text{group}}$$

where *VE*.*ims* is the vaccine efficacy defined using an immunological parameter alone from the immunogenicity subset participants, and *Ab* is the immunological parameter (usually measured one-month after vaccination). Different values for *t* are trialled until a value is found for which *VE*.*obs* will match *VE*.*ims* and this value of *t* is then retained as the threshold.[9, 15]

The above method for calculating thresholds, only considers clinical disease and levels of the immunological parameter as factors relevant to protection against disease. No other variables are included in calculations. The difference between vaccine and control groups is therefore assumed to be entirely mediated through the immunological parameter. Values of the immunological parameter above the threshold value are assumed to induce sterilizing immunity. This is a simplifying assumption as in reality, immunity is not mediated through a single immunological parameter such as circulating serum IgG, but also relies on other factors such as cellular immune function, and mucosal immunity. In addition, assumptions about sterilizing immunity may not be realistic for some immunological parameters.

### Population data

**Fig 1:**
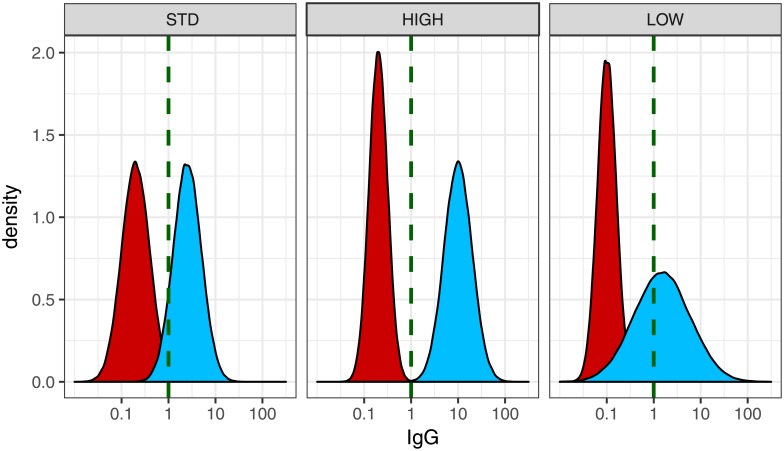
Simulated antibody distributions in vaccinated and control populations for three scenarios shown in Table 1. Red = control group, blue = vaccinated group. Green dashed line = antibody threshold that gives greatest level of protection. STD, HIGH, LOW: see Table 1 for definitions.

Individual participant data were simulated for a theoretical population of N = 400,000 people eligible for inclusion in a vaccine efficacy study, equally divided between vaccine and control groups. For each person an antibody level (*Ab*) for a hypothetical parameter assumed to be associated with protection against a suitable clinical endpoint (disease, infection, hospitalisation etc.), was simulated according to each person’s assignment to vaccine or control. An arbitrary value of 1 mcg/mL was set as the threshold antibody level at which the greatest protection against disease was reached (t*). Three vaccine scenarios were simulated to cover a range of likely scenarios; a) a vaccine that induces an antibody response which in most people is higher than the control group, however the antibody distributions overlap slightly (STD). This scenario is based on the immunogenicity results for pneumococcal conjugate vaccines reported in Siber et al [10], b) a vaccine that induces a very high antibody response which for all people is higher than the true threshold of protection (HIGH). This is a very optimistic scenario for an almost perfect vaccine. and c) a vaccine with low efficacy and a large proportion of non-responders (LOW), somewhat similar to the responses seen for rotavirus vaccines in low-middle income countries (Table 1 and Fig 1).

**Table 1:** Parameters for simulations of antibody distributions in vaccinated and control populations for three scenarios.

*Scenario*	*Vaccine antibody distribution* *(blue)*	*Control antibody distribution* *(red)*
STD: Vaccination produced a good antibody response however vaccine and control distributions still overlap a small amount	GM = 2.5 mcg/mL, m = 0.398, s = 0.3	GM = 0.2 mcg/mL, m = -0.699, s = 0.3
HIGH: Vaccine induces very high antibody response. Vaccine and control group antibody distributions are non-overlapping and all vaccinated participants have antibody above the threshold.	GM = 10 mcg/mL, m = 1.0, s = 0.3	GM = 0.2 mcg/mL, m = -0.699, s = 0.2
LOW: A large proportion of the vaccine group are non-responders. Vaccine and control distributions overlap substantially. Low vaccine efficacy.	GM = 1.5 mcg/mL, m = 0.176, s = 0.6	GM = 0.1 mcg/mL, m = -1.0, s = 0.2

GM = geometric mean, m = mean (on log_10_ scale), s = standard deviation of m; Antibody concentrations are simulated to be log-normally distributed, using a log of base 10.

**Fig 2:**
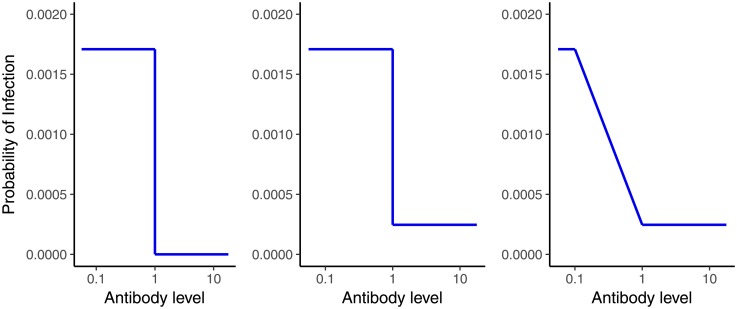
Three types of simulated relationships between antibody concentration and the probability of infection. A: Step function with antibody levels higher than the 1 mcg/mL threshold produced sterilizing immunity; B: Step function with antibody levels higher than the 1 mcg/mL threshold not producing sterilizing immunity; C: non-step function and non-sterilizing immunity.

Each person’s probability of disease (*p*) was determined according to their simulated antibody level, and the underlying relationship between disease and antibody as shown in Fig 2. Instances of disease were simulated from independent Bernoulli(*p*) distributions.

Three different relationships between antibody levels and disease probability were simulated. In the first scenario, the relationship between antibody and the probability of infection followed a step function and antibody levels higher than the threshold produced sterilizing immunity such that no disease cases were observed above this level (Fig 2A). This scenario aligns with the assumptions of the model and tests the performance of the model when all assumptions hold true. The second and third simulated scenarios test the performance of the method if the assumptions do not hold–that is, if the relationship between antibody and disease is not a step function or if sterilising immunity is not achieved. Thus, in the second scenario, the relationship between disease probability and antibody level was simulated as a step-function with antibody levels above the step threshold reducing disease probability substantially but not absolutely (Fig 2B). This represents a relative rather than an absolute threshold. In the third scenario a relative rather than absolute threshold was also investigated with the probability of disease simulated to decrease gradually as antibody levels increased, a situation likely to be closer to the true relationship between and antibody and disease than a step function (Fig 2C).

### Simulated trial level data

From the simulated population data, samples were repeatedly drawn to simulate enrolment of participants into separate trials. In total, 2700 trials were simulated with 150 trials for each of 18 scenarios (S1 Table). Trials were either of size 20,000 or 60,000 people and subsets of 1000 participants in each trial were randomly selected for inclusion in a simulated immunogenicity substudy. For the selected immunogenicity substudy participants, their antibody level was considered known (*Ab*), and for the non-selected participants, their antibody level was considered unknown (*Ab* = NULL). From each simulated trial population, *VE*.*obs* and *VE*.*ims* were computed and used to estimate *t* by the method described in Section 3.1.

## Results

**Fig 3:**
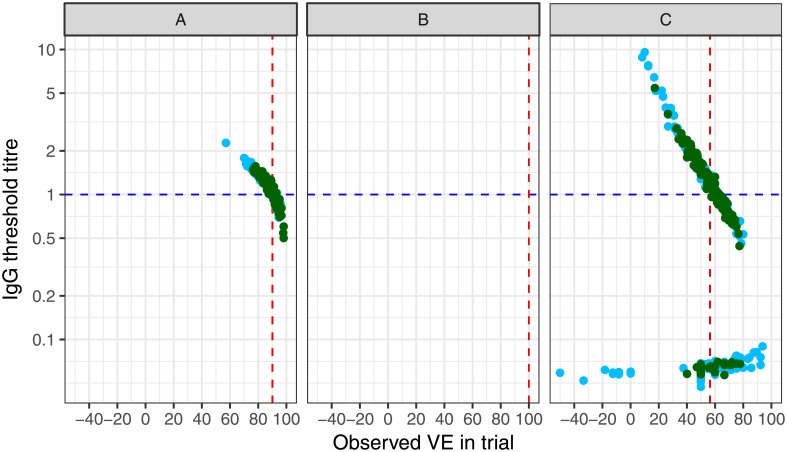
Estimates of the threshold of protection in 150 trials using three scenarios in Fig 1, and assuming that antibody levels above the true threshold produce sterilizing immunity in all people. Light blue dots = trials of size 20,000; dark green dots = trials of size 60,000; Blue dotted line = true threshold of protection (t*) of 1 mcg/m;, red dotted line = true vaccine efficacy. No estimates of the threshold could be obtained for the middle panel as all observed VE were 100%. Multiple values for t were obtained in 36 trials in the right hand panel, producing two distinct distributions. Simulation parameters shown in Tables 1 and S1.

The first set of results are presented in Fig 3 for the scenario in which the relationship between antibody and disease probability is a step function and antibody levels higher than the threshold are fully protective (Illustrated in Fig 2A). In Fig 3 each dot represents one trial, thus one estimate of *VE*.*obs* plotted against the calculated threshold of protection (*t*) from the immunogenicity substudy embedded within the hypothetical trial. The horizontal dotted line represents the true threshold of protection (t*) and the vertical dotted line indicates the true underlying *VE*. There was a decreasing relationship between *VE*.*obs* and *t*. Trials with estimates of *VE*.*obs* close to the true value, also had values of *t* close to the true value. However, trials with estimates of *VE*.*obs* that were lower than the true value, overestimated *t* and vice versa.

For the first scenario in which the simulated vaccine had good efficacy (Fig 1 STD & Fig 3A), estimates of *VE*.*obs* from simulated ‘small’ trials of 20,000 participants covered a wider range of values than *VE*.*obs* estimates from ‘large’ trials of 60,000 participants (Range of *VE*.*obs*: trials of 20,000, 57% to 100%; trials of 60,000, 76% to 98%). (Fig 3 STD). The median threshold computed was close to the true threshold, 1.04 (range: 0.69, 2.3) mcg/mL for small trials and 1.07 (range: 0.05, 1.6) mcg/mL for large trials.

In 28 small trials *VE*.*obs* was 100% and therefore a threshold could not be computed. In such situations, any value of antibody that is lower than the lowest value in the vaccine arm (i.e. all the antibody levels for the participants in the control arm) gave *VE*.*ims* estimates of 100% and fulfil the equations of the model. There are, therefore, almost as many valid values of *t* in each trial as there are participants in the control arm and no single solution can be obtained. Simulated trials with *VE*.*obs* of 100% are therefore not plotted.

Of the remaining 122 trials simulated in Fig 3 STD, a single threshold was obtained in 121 of these trials, however there were two solutions to the equations for one trial and thus two equally valid thresholds. This occurred when *VE*.*obs* was 62.5%. With a threshold of 2.012 mcg/mL the numbers of participants with antibody lower than the threshold in the vaccine and control groups were 184/500 and 500/500 respectively, giving *VE*.*ims* of 62.6%. At a threshold of 2.022 mg/mL the numbers were 188/500 and 500/500 giving *VE*.*ims* of 62.4%. Both thresholds give values of *VE*.*ims* that are equidistant from the *VE*.*obs* of 62.5% and thus both are equally valid solutions to the equation. In this example there is little material difference between the two thresholds obtained and such differences are likely to be less than any measurement error. The average of the two values could be retained as the final estimate.

When the vaccine had high efficacy (Fig 1 HIGH & Fig 3 HIGH) no cases of disease were observed in the vaccine arm thus each trial had *VE*.*obs* of 100% and no threshold could be obtained.

For the low efficacy vaccine (Fig 1 LOW & Fig 3 LOW) the median thresholds were 0.89 (range: 0.05, 9.6) and 1.11 (range: 0.06, 5.4) mcg/mL in small and large trials respectively. 16% and 8% of small and large trials respectively had more than one valid solution to the equations thus multiple estimates of *t* (Fig 3 LOW, S1 Table).

In Fig 3 LOW duplicates occurred at very different values of *t*. For example, when *VE*.*obs* was 60.0% and the threshold of 0.059 mcg/mL was used, the numbers below the threshold were 2/500 and 5/500 for vaccine and control groups respectively, giving a *VE*.*ims* of 60.0%. At a high value of *t* of 1.06 mcg/mL, the numbers below the threshold were 200/500 and 500/500, which also gives a *VE*.*ims* values of 60.0%. The difference between a threshold of 0.059 mcg/mL and 1.06 mcg/mL would not be considered an immaterial difference and so taking the average of the two is not a suitable way to resolve the problem of multiple solutions to the equations.

The second set of results are presented for the scenario in which the relationship between antibody and disease probability is a step function but antibody levels higher than the threshold are not absolutely protective (illustrated in Fig 2B).

**Fig 4:**
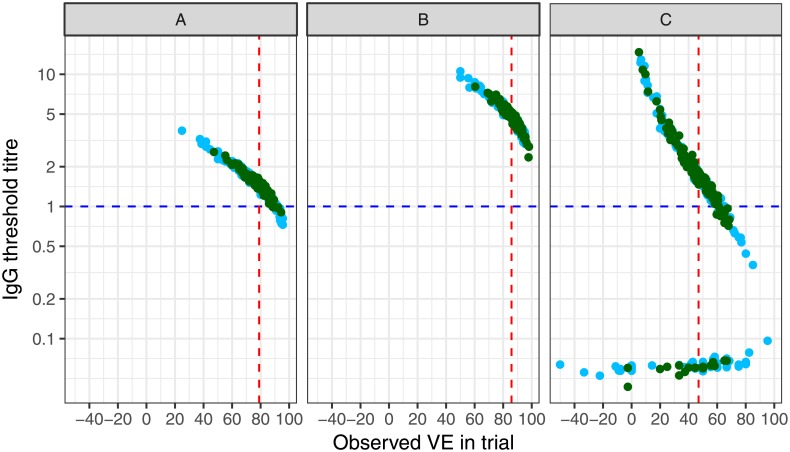
Estimates of the threshold of protection in 150 trials using three scenarios in Fig 1, assuming a step function in the relationship between antibody and disease, and that antibody above the threshold is not fully protective in all people. Light blue dots = trials of size 20,000; dark green dots = trials of size 60,000; Blue dotted line = true threshold of protection (t*) of 1 mcg/m;, red dotted line = true vaccine efficacy. Multiple values for t were obtained in 22 trials in the right hand panel, producing two distinct distributions. Simulation parameters shown in Tables 1 and S1.

For the scenario in which the simulated vaccine had good efficacy, the median thresholds were 1.47 mcg/mL (range: 0.73–3.74) and 1.45 mcg/mL (range: 0.90–2.59) for trials of size 20,000 and 60,000 respectively (Fig 4 STD, S1 Table). When the vaccine produced high antibody levels that did not overlap with the distribution of antibody in the control group (Fig 1 HIGH). The median threshold was more than 5 times larger than the true value (Median 5.43 mcg/mL, range: 3.09–16.8; and median 5.40 mcg/mL, range: 2.62–10.3, for small and large trials respectively) (Fig 4 HIGH). When the vaccine had poor efficacy (Fig 1 LOW) median thresholds were inflated and spanned a large range of values; 1.59 mcg/mL (range: 0.04–14.7) and 1.43 mcg/mL (range: 0.77–5.9) for the small and large trials respectively (Fig 4 LOW). In 22 of the simulated trials for the scenario displayed in Fig 4 LOW there were multiple solutions to the equations and thus some trials had a very low threshold value as well as a threshold value similar to the main distribution of estimates of *t*.

**Fig 5:**
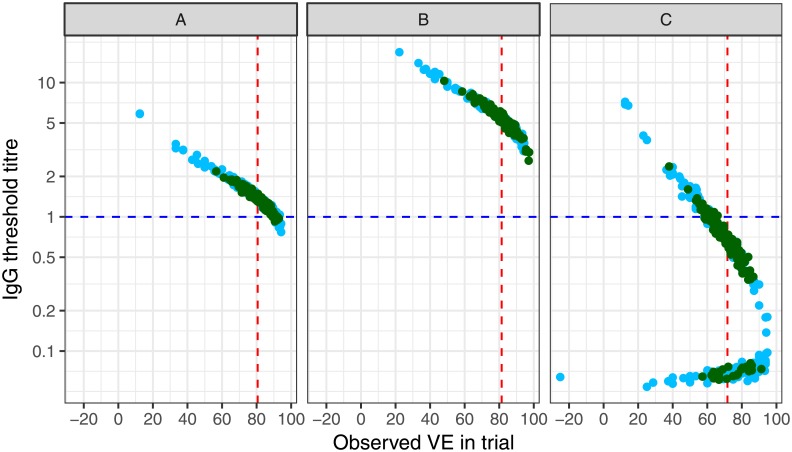
Estimates of the threshold of protection in 150 trials using three scenarios in Fig 1, assuming a graduated threshold (non-step function) in the relationship between antibody and disease, and that antibody above the threshold is not fully protective in all people. Light blue dots = trials of size 20,000; dark green dots = trials of size 60,000; Blue dotted line = true threshold of protection (t*) of 1 mcg/m;, red dotted line = true vaccine efficacy. Multiple values for t were obtained in 46 trials in the right hand panel, producing two distinct distributions. Simulation parameters shown in Tables 1 and S1.

When a more realistic relationship between antibody and disease was used for simulations which incorporated a gradual decline in disease probability with increasing antibody levels, (Fig 2C) the pattern of threshold estimates was similar to that in which there was a hard step function (Fig 5A and 5B). In Fig 5C which displays the scenario in which there are many non-responders, threshold estimates were lower than the true value in the majority of cases.

**Fig 6:**
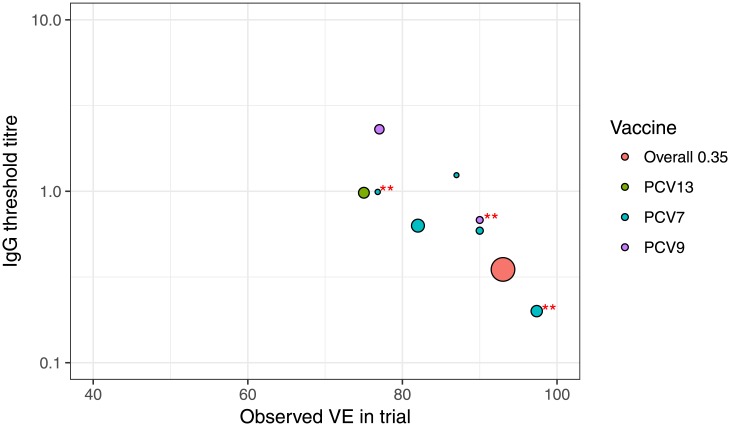
Published estimates of overall anti-capsular IgG thresholds of protection against invasive pneumococcal disease from vaccine efficacy trials and indirect cohort studies. Estimates of anti-capsular IgG thresholds of protection against invasive pneumococcal disease from individual studies of PCV7, PCV9, and PCV13 from refs [1–3, 10, 11, 16, 17]. Overall: the threshold of 0.35 mcg/mL derived from three studies combined (**) [1–3], and used for vaccine licensure. The size of each circle is proportionate to the inverse of the standard error of the observed vaccine efficacy estimate.

Published estimates of overall pneumococcal conjugate vaccine correlates of protection defined from vaccine efficacy studies using the methods described herein are displayed in Fig 6. A clear relationship between the estimated thresholds of protection and the observed vaccine efficacy estimates can be seen in the published studies that mimics the relationship seen in the simulated data. Higher values of *VE*.*obs* produce lower threshold values and the reverse is also true. The similarity between published and simulated estimates shows that the simulations provide a true depiction of the findings of current research.

## Discussion

Calculating thresholds of protection by matching vaccine efficacy estimates from studies measuring clinical disease and embeded substudies with immunological endpoints, produces estimates which can be highly inflated. Our simulations show that when a highly efficacious vaccine induces a distribution of antibody values that is higher than the control group, and higher than a true level of protection, the model will either produce substantially inflated threshold estimates, or if the observed VE is 100%, then no threshold at all. This could lead to the situation whereby a true threshold that is fully protective might exist at e.g. 1 mcg/ml, but the threshold computed based on a trial of a highly efficacious vaccine is greatly inflated (e.g. 5 mcg/mL). Such an over-inflated threshold estimate would have the opposite outcome to that desired, by preventing other vaccines being licensed. For example, a new vaccine that induced on average 3 mcg/mL of antibody at one-month post vaccination would be considered not sufficiently immunogenic for licensure under this paradigm even though the true threshold of protection was 1 mcg/mL and the new vaccine would have be highly protective if tested in the field.

For both pneumococcal and meningococcal polysaccharide-protein conjugate vaccines the defined and accepted thresholds of protection have been shown to have limitations. Clear instances of disease occurring in the presence of high antibody levels have been observed [18] as well as serotype-specific differences in protective levels for pneumococcal vaccines.[11, 19, 20] However, this method has been used to establish licensure for more than one vaccine that has subsequently proven to be effective and thus defining the original threshold was a useful process which led to successful vaccine development.[14, 21–23]

In the case of pneumococcal conjugate vaccines, the trials used to estimate the accepted threshold of 0.35 mg/mL observed high efficacy values.[1–3] Our simulations show that when *VE*.*obs* is high the threshold value that results is low and may be lower than the true threshold. It is possible therefore, that the threshold of 0.35 mg/mL for pneumococcal vaccines is an underestimate of the true threshold value.

An assumption that a threshold of absolute protection does exist is more reasonable for some vaccines than for others. We have previously modelled the relationship between pneumococcal carriage infection and antibody response to vaccination in infants, revealing that a point at which the probability of carriage becomes negligible does exist for most serotypes and the levels vary between them.[20] Thus for some (in particular polysaccharide capsular conjugate) vaccines a true threshold of absolute protection may exist. However, for other organisms the relationship between antibody and infection may be different due to different underlying immunological processes, such as for live attenuated viral vaccines or intracellular pathogens. This can be observed for infections with *Salmonella* Typhi in which an increasing level of anti-Vi-IgG is associated with increasing protection against disease, yet challenge studies and seroefficacy estimates reveal that there is no observable threshold of Vi-IgG whereby protection becomes absolute.[24] For pathogens in which data suggest absolute thresholds are implausible, our simulations reveal that attempting to calculate thresholds of protection will lead to inflated values in many scenarios, particularly when the vaccine induces a good antibody response. This is not surprising as such situations violate the assumption underpinning the model. Unless there is strong evidence for absolute threshold of the sort illustrated in Fig 2A, researchers should not report a single threshold antibody level as a correlate of protection, lest it turn out to be biased as demonstrated by these simulations, and subsequently proceeds to become an accepted value.

The challenge for vaccine development for pathogens for which an absolute threshold is not plausible, is to find a method to compare and test the protective efficacy of new vaccines without requiring large field studies.

When an absolute threshold is unlikely to exist, vaccines can be compared using relative measures. For example, Vi-polysaccharide typhoid vaccines have an estimated 2-year efficacy of 59% (95%CI 45–69%) against typhoid fever [25], and typically illicit antibody responses 6 weeks after vaccination of between 300 and 500 EU/mL when measured using the VaccZyme ELISA kit.[26] The Vi-tetanus toxoid typhoid conjugate vaccine induces higher antibody levels in the range of 1000 to 1500 EU/mL in an endemic setting [26], and although effectiveness in the field has not yet been demonstrated, trials are underway, and a seroefficacy study has suggested the vaccine may be 85% protection.[27] Rather than seek to determine an absolute threshold of protection when such a thing may not exist, future vaccines can be compared with licensed vaccines with known efficacy by comparing the proportion of participants above a median antibody level. Since the variation in a proportion is maximised at 50%, this value (equivalent to the median) may be the most useful and the most conservative cutpoint to use for clinical trial comparisons. It does not matter to the clinical trial results, that this cutpoint does not equate to a level of absolute protection and it does not need to be considered a threshold of protection, it is simply a useful cut point for comparing two arms of a randomised trial. Thus for Vi polysaccharide typhoid vaccines, if the proportion of participants with Vi-IgG above 400 EU/mL is similar in both arms of a trial then the vaccines would be expected to be similarly effective in the field and it does not matter than the value of 400 EU/mL is not a protective threshold. For conjugate vaccines, a value of 400 EU/mL is too low as almost all participants will have antibody above this cutpoint and thus a value of 1250 EU/mL would be more appropriate for comparisons. Comparisons of vaccines in which 99% of participants have antibody above a certain cutpoint in both arms of the trial do not provide enough information to discriminate between the vaccines and indicate that the cutpoint used for comparisons is too low. Comparisons of the proportions of participants above a cutpoint are similar to a comparison of geometric means when the cutpoint is set at a value equivalent to the expected median in the control arm.

For all pathogens, it is important to determine whether an absolute threshold of protection is likely to exist. This can be achieved by examining the relationship between an immunological parameter and the probability of disease when modelled appropriately using logistic or generalised additive models, or other appropriate probabilistic models. This has been done successfully for pneumococcal carriage studies.[19, 20] However, these models require suitable numbers of events to appropriately characterise the shape of the relationship and cannot therefore be used when modelling rare events such as blood culture positive enteric fever or invasive bacterial diseases. In these situations, models based on serological methods for detecting disease events show greater promise. Clinical events may be rare in field vaccine trials but serologically-defined events are more common and these can be used as endpoints in smaller immunogenicity trials to estimate vaccine sero-efficacy and define thresholds of protection.[20, 27]

### Conclusions

Current methods for determining a threshold of protection for use in vaccine development can produce inflated thresholds, particularly in situations where a vaccine induces high levels of antibody, or when the threshold is not indicative of sterilizing immunity. Absolute thresholds of protection are unlikely to exist for many pathogens and in these situations we recommend using alternative statistical methods, such as modelling of serologically defined infections to suitably characterise the shape of the relationship between an immunological parameter and the probability of infection.

## Supporting information

S1 Table Simulated scenarios and results. (DOCX)
