# Supplementary material for: Computing threshold antibody levels of protection in vaccine clinical trials: An assessment of methodological bias
Source: PLoS One. 2018 Sep 7;13(9):e0202517. doi: 10.1371/journal.pone.0202517 (PMC6128451; doi:10.1371/journal.pone.0202517)
Supplement: S1 Table — (DOCX) [file pone.0202517.s001.docx]

Table S1 Simulated scenarios and results

| Relationship between Ab and disease | Vaccine scenario | True VE | Trial size | Immuno substudy size | Number of trials simulated | Range of values for VE.Obs in 150 trials | threshold (t)  (mcg/mL)  median [range] | Number of trials with multiple values of t | Simulation results shown in |
| --- | --- | --- | --- | --- | --- | --- | --- | --- | --- |
| Sterilizing immunity – Fig 2A | Fig 1 STD | 90.1% | 20000 | 1000 | 150 | 57 − 100% | 1.04 [0.69, 2.27] | 1 | Fig 3A |
|  |  | 90.1% | 60000 | 1000 | 150 | 76 − 98% | 1.07 [0.50, 1.57] | 1 | Fig 3A |
|  | Fig 1 HIGH | 100% | 20000 | 1000 | 150 | 100 − 100% | - |  | Fig 3B |
|  |  | 100% | 60000 | 1000 | 150 | 100 − 100% | - |  | Fig 3B |
|  | Fig 1 LOW | 56.4% | 20000 | 1000 | 150 | - 50 − 94% | 0.89 [0.05, 9.58] | 24 | Fig 3C |
|  |  | 56.4% | 60000 | 1000 | 150 | 17 − 78% | 1.11 [0.06, 5.43] | 12 | Fig 3C |
| Step function threshold – Fig 2B | Fig 1 STD | 79.0% | 20000 | 1000 | 150 | 25 − 96% | 1.47 [0.73, 3.74] | 4 | Fig 4A |
|  |  | 79.0% | 60000 | 1000 | 150 | 47 − 94% | 1.45 [0.90, 2.59] | 0 | Fig 4A |
|  | Fig 1 HIGH | 85.8% | 20000 | 1000 | 150 | 50 − 96% | 4.93 [2.97, 10.53] | 0 | Fig 4B |
|  |  | 85.8% | 60000 | 1000 | 150 | 61 − 98% | 4.83 [2.35, 8.05] | 1 | Fig 4B |
|  | Fig 1 LOW | 47.0% | 20000 | 1000 | 150 | - 50 − 95% | 1.29 [0.05, 12.9] | 15 | Fig 4C |
|  |  | 47.0% | 60000 | 1000 | 150 | - 3 − 69% | 1.59 [0.04, 14.7] | 7 | Fig 4C |
| Graduated threshold – Fig 2C | Fig 1 STD | 80.6% | 20000 | 1000 | 150 | 13 − 94% | 1.43 [0.77, 5.9] | 5 | Fig 5A |
|  |  | 80.6% | 60000 | 1000 | 150 | 57 - 93% | 1.37 [0.92, 2.2] | 3 | Fig 5A |
|  | Fig 1 HIGH | 81.4% | 20000 | 1000 | 150 | 22 – 94% | 5.43 [3.09, 16.8] | 6 | Fig 5B |
|  |  | 81.4% | 60000 | 1000 | 150 | 48 − 97% | 5.40 [2.62, 10.3] | 2 | Fig 5B |
|  | Fig 1 LOW | 71.7% | 20000 | 1000 | 150 | - 25 − 95% | 0.31 [0.05, 7.2] | 41 | Fig 5C |
|  |  | 71.7% | 60000 | 1000 | 150 | 38 – 91% | 0.64 [0.06, 2.4] | 5 | Fig 5C |
| **Total number of trials simulated** | |  |  |  | **2700** |  |  |  |  |
